# A phenomenographic study of students' conception of learning for a written examination

**DOI:** 10.5116/ijme.5513.0eec

**Published:** 2015-03-30

**Authors:** Desiree W. Edström, Niklas Wilhemsson-Macleod, Michel Berggren, Anna Josephson, Carl-Fredrik Wahlgren

**Affiliations:** 1 Dermatology Unit, Department of Medicine Solna, Karolinska Institutet and Karolinska University Hospital, Sweden; 2 Department of Otorhinolaryngology, Karolinska University Hospital, Sweden; 3 Department of Neuroscience, Karolinska Institutet, Sweden

**Keywords:** assessment, curriculum change, integration, phenomenography

## Abstract

**Objectives:**

We investigated students' conception of learning for an examination in internal medicine, infectious diseases and dermatology-venereology, in three separate examinations versus a single integrated one.

**Methods:**

The study was carried out during a curricular change, with one cohort belonging to a new integrated examination and the other to the former non-integrated examination. Forty-eight interviews were carried out among medical undergraduates regarding the role of the examination in the learning process. The interviews were analyzed according to the phenomenographic approach to identify the students' conception of learning.

**Results:**

The learning approaches could be categorized in 47 of the 48 students into 4 major groups: application directed, holistic, comprehensive and tactical memorizing learning. The result indicated that comprehensive learning was the most common approach among students following either examination-form; tactical memorizing learning was more prevalent among students following the non-integrated examination and holistic learning was applied more frequently among students following the integrated examination. Nine of the 47 students changed their approaches over time, the majority switching to a compre-hensive approach. No significant gender difference was observed.

**Conclusions:**

Comprehensive learning was the most common strategy employed and students who changed during the course most often switched to this. However, only a minor change in approach was observed after a switching to an integrated examination, i.e. it takes more than just an integrated examination to change the stu-dent's conception of learning.

## Introduction

There is a changing focus in healthcare which calls for a curriculum change, with a shift from the cure of disease, episodic care, and anecdotal care to the preservation of health, continuous and comprehensive care and evidence-based medicine.^1^ Many universities modify their curriculum to stimulate integrated learning by setting integrated courses and integrated examinations. The medical programme at Karolinska Institutet conducted a curriculum change in 2008. Some major reasons for this were to add primary care across the whole programme, to launch clinical electives as well as a 20-week individual scientific project. Another purpose was to stimulate integration between subjects, both vertically and horizontally. However the courses were still separate with only a few days to integrate the subjects. The examination was the main change towards employing integration for the students. This curriculum change gave us the unique opportunity to analyze potential changes in the students' conception of learning in preparation for a written integrated examination in undergraduate medical training.

It is well known that assessment drives learning and influences students' study habits substantially.^2^^-^^8^ Assessment can be performed in many ways depending on its role in the curriculum. Medical school examinations in their present form serve several purposes.^9^ In general, these comprise a measure of the progress in theoretical knowledge, to integrate and finalize a certain portion of course work, and finally to motivate and rank students. Assessment is often viewed as a frightening obstacle at the end of a course with subsequent negative effects on learning.^10^ Clinical performance of medical students does not seem to correspond with the performance in final written examinations.^11^ Indications of an uncertain congruence between results on written assessment and long-term understanding are reported.^12^ Frequent use of old examination material leading to a superficial approach to learning is another problem,^13^^, ^^14^ and medical students are at a particularly high risk of developing such a study practice.^15^

Van der Veken et al studied the longitudinal changes in students' learning pattern parallel to the implementation of an integrated medical curriculum.^16^ They used the inventory of learning styles (ILS), a questionnaire of 126 items. They saw an impact on learning processing strategies with a holistic approach relating more parts of the subject to each other and to earlier acquired knowledge. The limitation of the ILS research instrument is that it does not reflect the actual learning behavior, but rather the students' conception about their own learning pattern as pointed out by others.^17^^,^^18^ Instead of using a fixed questionnaire we wanted to study the students' conception of learning with phenomenograpy, an unprejudiced method. Our assumption was that an integrated examination would help the students to see the context and affect their approach to learning for the examination. Besides, to the best of our knowledge we were not aware of any study investigating the students' conception of learning when the integration was restricted to making only the final written examination integrated.

The aim of the study was to investigate the medical students' conception of learning for a written examination and to analyze if and how these changed when three separate written examinations were integrated into one final overall written examination.

## Methods

### Study design

The Swedish Medical Programme consists of five and a half years of studies divided into eleven semesters. At our university the former curriculum comprised separate courses in internal medicine (20 weeks), dermatology-venereology (4 weeks) and infectious diseases (4.5 weeks). These were studied and examined independently. In January 2008, a new curriculum was launched with the aim of integrating courses, reducing fragmentation and providing space for a compulsory, 20-week scientific project and several elective courses. This meant that clinical pharmacology, primary care, geriatrics, scientific and professional development were added to internal medicine, dermatology-venereology and infectious diseases, forming a new 32-week course called Clinical medicine. In this course, the new curriculum was implemented principally by introducing an integrated final examination.The course was mainly located at four hospitals in Stockholm affiliated with several local primary care centers and geriatric hospitals. Since some parts of the course were held in several hospitals/units (e.g. geriatrics, primary care), whereas others were given at only one (e.g. dermatology-venereology) or two (e.g. infectious diseases) sites, it was not logistically possible to fully physically integrate them, with the exceptions of certain seminars, discussion groups and the final examination.

In the former curriculum, the students in internal medicine had three written tests with short answer open-ended questions and a written final examination covering the whole content of the course. The course in dermatology-venereology included a written half-time test with twenty short answer questions and a final written examination including twenty photos of skin diseases to be diagnosed, fifteen short answer open-ended questions for preclinical and clinical theory and clinical management, as well as one essay question. The course in infectious diseases comprised a half-time test containing ten written short answer questions and a final written examination with approximately thirty short answer, open-ended questions.

The new integrated final examination consisted of eighty open-ended, short answer questions and two modified essay questions (MEQ) giving a total score of 100. The MEQs were fully integrated whilst the short answer questions were less often integrated.

To be able to compare the examination results from the two curricula, the old curriculum results were calculated as a percentage of the total score of the three final examinations by adding the scores from internal medicine, dermatology-venereology and infectious diseases.

The board of Ethics at Karolinska Institutet reviewed our study and stated that no ethical approval was required for this kind of study. Oral informed consent was obtained from the participants.

### Participants

Two cohorts were identified among the students who had passed the final examination(s), one with 52 students from the old curriculum (the last course given in autumn 2007) and one with 57 students from the new (the second course, given in autumn 2008). These students represented all students who had passed the examination(s) in two hospitals, Danderyd's hospital and Karolinska Solna. The students were, in order to avoid inclusion bias, stratified into three subgroups according to their results in the final exam (low, medium and high score), to account for possible differences in conception of learning depending on examination result. The students were randomly selected with each subgroup approached and invited to participate by telephone calls. We recruited 8 (4 male and 4 female students from each subgroup and cohort, forming 6 groups with 8 students in each group. The interviewers did not know to which score group each student belonged. The median age was 25 years (age range 21-37) for the students from the old curriculum and 24 years (age range 22-31) for the students from the new.

### Data collection

We collected data through individual, semi-structured interviews, allowing for broad descriptions of the topic. The interviews were conversational in style allowing some flexibility for each student to elaborate on their own experiences while still providing stringency. An interview guide was used, and the questions covered how the students perceived of their studies, the role of the examination(s) in their studies, and how they experienced the examination(s). The interviews were performed by two of the authors (DWE and NW-M), who had not been involved in teaching the current students. The interviews lasted between 45 to 60 minutes and were carried out in a neutral room containing only a table and chairs at the Karolinska University Hospital, Solna. Two pilot interviews were performed. The written transcripts of the two interviews were evaluated and discussed within the group in order to adapt the questions to fit the purpose and to ensure the researchers were confident in their task. The remaining interviews were performed 2-3 months after the completed course. The interviews were carried out in Swedish and the quotes that are referred to in the result section have been translated into English.

### Procedure and data analysis

The interviews were recorded, transcribed and printed. The written transcripts were analyzed according to the phenomenographic approach. Phenomenography is an approach for identifying and describing qualitative variation in individuals' experience of their reality.^19^ As a qualitative method, phenomenography seeks to reveal the variation of conceptions held by the participants regarding the phenomenon and to express these in distinctive, complete and hierarchical categories.^20^ In phenomenographic research the term conception is used to refer to people's ways of experience a specific aspect of reality, presented in the form of categories.^19^ We choose a phenomenographic approach to study the students' conceptions, as such an approach had previously been used in our research group.^12^

Three of the authors read the complete interviews separately. Bearing elements in the transcripts were discerned and discussed via phenomenograpic analysis. Grouping of different elements was made possible through identifying similarities and differences using qualitative data analysis computer software (NVivo 8, QSR International Limited, Southport, UK). The resulting clusters formed the basis of the four categories presented in the result section. Thereafter, all transcripts were re-read and the three (DWE, NW-M, MB) authors' individual categorizations were discussed, compared and adjusted through negotiated consensus before arriving at the final set of categories.^21^ This process was repeated several times until the core meaning of each category was clarified and defined against neighbouring categories.^22^

## Results

### Learning conceptions

The analysis yielded four categories representing different approaches in the students' own conception of learning for the final written examination(s). These categories are presented below with quotes to elucidate their meaning.

### Application directed learning

This category is based on learning for life and is characterized by the student's own responsibility for learning, which goes beyond the examination. The future profession and the knowledge applicable in that setting are the ultimate aims of their studies. A good result on the examination does not guarantee that one will become a good doctor and is thus regarded to be less important in it-self. Practical application of knowledge performed in everyday clinical work is to the fore: learning during every day clinical work is considered more important than the final examination. The approach towards the examination is problematized in relation to the knowledge that it should measure. If you have a thorough understanding, it shows in the examination, but passing does not necessarily guarantee a sound understanding.

"Examination of any kind is really just necessary for the teachers to verify that you grasp the content of the course, but for me it is not the main thing with a course but for me the main aim of a course is not to pass the examination, but to possess the knowledge when you are a graduated doctor." (23-year-old male student from the old curriculum, scoring high at the final examination)

"I have a constant thought that I want to know this, this disease or the way to investigate it. I want to have the knowledge of it when I meet the patient, and the whole time there is a very clear goal why I have to learn certain things." (24-year-old female student from new curriculum, scoring high at the final examination)

"I think most people would say the exam plays an extremely small role, but this might not be always true. It plays a certain role if I am totally uninterested in the course, because then it (the examination) becomes a compulsion which can be a drive in such cases when I am not interested in the subject, but this situation is extremely rare." (25-year-old female student from the new curriculum, scoring average at the final examination)

### Holistic learning

This category is focused on an interconnected experience of knowledge from various aspects and disciplines in medicine, integrating previously separated aspects of knowledge. The assessment as well as the necessary revising has an integrative function. Connections are made between sections of knowledge creating an understanding, which is qualitatively different from earlier experiences of the same knowledge. The examination is a goal in itself, since it is assumed to secure a good understanding. It could also pertain to using different patterns of thought and alternative approaches to a medical problem.

"For obvious reasons you study more intensely for an exam, because you try to bring together everything you have learned, link things and see them in a new light." (27-year-old female from the old curriculum scoring high at the finale examination)

"It plays a big role; I believe that if we did not have an exam I would probably not be cramming as much. It is not the best method to simply revise before the exam but you learn something anyway and you tie up the bag and get an overview of the course which you have read." (28-year-old male from the old curriculum, scoring average at the final examination)

"To try to get an overall picture of how to deal with, perhaps, combinations of different states, priorities etc., and to somewhat tie it all together, that is somehow the goal." (24-year-old male student from the old curriculum, scoring high at the final examination)

"It's the exam that at the end of the course makes you sit and repeat everything. Most of it you know quite well anyway, but the last details and the overall perspective tend to fall into place when you repeat everything. So the exam is more about connecting all the different subsections." (22-year-old female from the new curriculum, scoring average at the final examination)

"I do a lot of mind maps. I take data from what I am taught and then processes it by myself... it feels good if you can tie it together with the exam, the revision for the exam was a way to put it all together." (24-year-old male student from the new curriculum, scoring average at the final examination)

### Comprehensive learning

Knowledge is sorted under discrete and condensed headlines, which are not given a new relation during revision. Neither revision nor assessment brings about an understanding that was not already established. The student is eager to learn and applies a superficial learning approach, memorizing disintegrated data without seeking in-depth understanding of their relationship. The examination is perceived as an important goal in itself, but emphasis is not on understanding, rather on memorizing what they regard as important knowledge.

"It is about repeating things. The more times you repeat, the better you know something and especially before the final examination after such a long course as medicine where you have to repeat everything from the entire course." (25-year-old male student, scoring high at the final examination)

"It (the examination) is of some importance, it forces you to repeat things and that is good. The other good thing is that I set aside time to actually repeat things." (24-year-old female student from the old curriculum, scoring high at the final examination)

"I don't think that I would have learned as much if we hadn't had any exams, because then I wouldn't have read everything once again. I think it feels a bit like a whip at my back which forces me to repeat it all once again." (26-year-old female student from the old curriculum, scoring low at the final examination)

"It (the examination) plays a pretty big role, I think. Because you have to revise the knowledge you have gained over the course once again and you have to repeat the most important knowledge... I read old exams which I think is good. I usually look at the old questions and if I can answer them, then I consider myself to know the subject." (25-year-old male student from the old curriculum, scoring average at the final examination)

"...honestly, I read the old tests... one can manage basically by reading the old tests or exams only...if I pass an examination with a good score, then I believe I will have something useful to take with me for the future, it is a proof that I have learned something; it is important that it (the examination) goes well, and therefore I study a lot beforehand." (24-year-old female student from the new curriculum, scoring average at the final examination).

"The exam is of great importance, bigger that I would like it to be. I don't really want to be one who is only studying to pass the exams, but rather someone who likes to study to become a good doctor. But it becomes a strategy of survival to pass the exams, and to assume that what you know at time of the exam is also what you need for the future when you have finished medical school." (22-year-old male student from the new curriculum, scoring high at the final examination)

"...if it is so that you haven't passed the exam, it's because you didn't study the in the right way or study the right thing. The exam is important; it is important symbolically, it's some kind of confirmation that you have done what you should have and now you are ready for the next step." (25-years-old male student from the new curriculum, scoring low at the final examination)

"For me the mastering of knowledge is very important, and both the tests and the final exam, give you a reason to repeat the knowledge you have. It's good to have some kind of strategy in the reading and I believe that the old exams usually prove what will be focused on in the coming exam." (31-year-old female student from the new curriculum, scoring low at the final examination)

### Tactical memorizing learning

The examination is perceived as a necessary evil and tactical memorizing is the most employed way to pass the exam. Neither a good nor a bad result is thought to lead to a long-lasting understanding or the competence required from a medical doctor. The examination is from a technical point of view naturally an important part of the course, but, in view of further medical training, irrelevant when it comes to learning. Theoretical studies are not given priority and medical competence is assumed to be acquired later, in a future problem-based working situation.

"It is not a receipt as to what I have learned; I don't think it is, not in my experience. But it is something that should be done and hopefully you end up passing the examination." (31-year-old female student from the old curriculum, scoring average at the final examination)

"Honestly, I must say that when I read, I actually read to pass the exam, even if it should not be that way; rather, you should read to understand the subject better, but that comes after a while, the exam is mostly a check of one's knowledge. It is a guarantee to the next step and much of a stress factor and so on." (24-year-old male student from the new curriculum, scoring low at the final examination)

"I believe there is a reason for the questions in the exam that I need a 'kick in the butt' to sit down and really revise thoroughly. The exam has a good function in that way, but it's also a stressful factor also to find motivation to cope with the exam. It becomes a dominance of coping rather than actually learning what you are supposed to learn." (26-year-old female student from the new curriculum, scoring average at the final examination)

"Not very much (what role the examination played), I must say; it is clear that in a way it does when you revise and you can link everything together, but that is not what has given me the lasting impression. The lasting impressions are bed-side and my own patients. It is rewarding to repeat old written exams to read how the questions are asked and what answers are requested and also that you get to repeat the material. It's so boring reading a book or your own notes from the lecture, and therefore I believe that it is good to read old exams so that you have an overview." (25-year-old male student from the old curriculum, scoring high at the final examination)

### Distribution of conceptions of learning at the end of the course

A comprehensive learning was the most common approach independent of whether the examinations were integrated or not. However, the proportion of tactical memorizing was somewhat higher in the old curriculum while the proportion of holistic learning was higher in the new curriculum with an integrated examination.

At the end of the course the number of students from the old and new curriculum was 2 and 2, respectively, in category application directed learning, 3 and 6 in holistic learning, 11 and 12 in comprehensive learning and 8 and 3 in tactical memorizing learning. The learning approach of one of the students from the new curriculum could not be categorized, due to far too vague answers. Some students (9 of 47, 19%) changed their conception during the time of the course. One student changed from application directed to holistic learning, and two students from application directed to tactical memorizing learning. Four students changed from holistic learning to comprehensive learning and one student changed the opposite way. One student changed from tactical memorizing learning to comprehensive learning. Comprehensive learning was the largest category, and showed the largest net influx of students over time. The changes in conception of learning over time were overall the same for both the old and the new curriculum. No significant gender differences were observed. We were unable to compare the overall results of the two cohorts, due to the examinations being different in nature (integrated versus non-integrated).  

## Discussion

Comprehensive learning was the most common strategy and students who changed during the course most often switched to this. There was a tendency to a more holistic and less tactical memorizing conception of learning among the students' studying for the integrated examination. However, only minor change was observed after a change to an integrated examination, i.e. an integrated examination in itself was not enough to change the medical students' conception of learning.

Learning has several components, namely cognitive processing strategies, metacognitive regulations strategies, conceptions of learning and learning orientations.^23^ The understanding and application of knowledge is crucial to learning.^24^ Three dominant approaches to learning have been described: deep approach (seeks after a meaning, relates new ideas to previous knowledge and is evidence critical), strategic approach (organized studying, good time management and a desire for high achievement) and surface approach (the student memorizes without understanding).^13^^, ^^25^^, ^^26^ Our results throw a different light on conception of learning as the study was performed with specific reference to a forthcoming examination. Our category application directed resembles deep approach, while category tactical memorizing learning echoes surface approach. The categories holistic learning and comprehensive learning both contain aspects of deep and surface approach.

We have studied the conception of learning for examination(s) in undergraduate medical students by interviewing them and applying a phenomenographic methodology. Phenomenographic research is about revealing the possible ways people understand a certain phenomenon. We analyzed the interviews based on the participants' perceptions and free from influence from prior findings, an approach discussed by other groups.^27^^,^^28^ Our findings are consistent with previously described observations.^29^ A comprehensive learning was the most common approach independent of whether the examinations were integrated or not. However, the proportion of tactical memorizing learning was somewhat higher in the old curriculum while the proportion of holistic learning was higher in the new curriculum with an integrated examination. This might indicate that an integrated examination gave another opportunity to repeat the complete material from the course, and that tactical memorizing learning in our study was a tedious approach to learning extensive material.

Over the time of the course we observed a shift in learning approaches among the students. These changes were largely similar, qualitatively (shift of category) as well as quantitatively (number of students), for both the old and the new curriculum. On the one hand the shift tended to go from a more application directed and holistic approach that focuses on understanding and analytical thinking, in favour of a more comprehensive/ tactical memorizing approach that focuses on memorizing without further analysis. On the other hand, the shift tended to increase the perceived importance of the examination as an external motivator for learning. This seems to highlight a shift in the students' focus from internal to external motivational factors, where passing the examination rather than learning becomes the most important goal of studying the closer to the examination they get. Overall, an approach to learning for the future profession (application directed learning) tends to be abandoned for a more mnemonic approach to learning (comprehensive learning) as the students get closer to the written examination. This shift fails to meet one objective of the curriculum change, which aims at achieving higher Structure of Observed Learning Outcome (SOLO) levels in the student's quality of learning, with less uni-structural or multi-structural level and more relational or extended abstract stages.^30^ Integration as performed in the present curriculum change does not per se imply higher SOLO levels.

It has been suggested that an effective method to change students' approach to learning is to alter the form of assessment. This has become known as the backwash effect in learning. Backwash implies that students adapt their learning depending on what they think will be covered in the assessment.^31^^,^^32^ Backwash could account for a shift towards a more superficial learning approach in a tactical medical student. One such superficial learning tactic often employed by the students includes the use of old written examinations, available on line, whereby students quickly recognize frequently recurrent and sometimes even unaltered questions. Such techniques would give students a clear advantage in the examination, but do very little for their conceptual understanding. As the time of the examination gets closer, students find it increasingly important to pass and so their internal motivation to learn is replaced with an external motivation to pass the test. Students therefore employ more time-efficient ways of passing the examination and rely more on superficial learning approaches. This was confirmed in our study and was valid for students both in the old and new curriculum. An integration of the examination seemed to have little or no effect on students' conception of learning, especially if the questions are not fully integrated and the students are aware of this prior to the examination.

### Limitations

It was not possible to examine how the different learning approaches affected the results on the examination. We divided the two cohorts into three groups (low, medium and high score), but we were unable to compare the overall results of the two cohorts due to the examinations being different in nature (integrated versus non-integrated). The interviews were carried out by two researchers, one who was very familiar with phenomenography and one who was not. This might influence the interviews and the result. However, two pilot interviews were made to minimize this bias. The interviews were performed two to three months after the final examination, and the gap in time might also have resulted in the students forgetting how they went about their studies for the examination(s). This might influence the individual student's reflection but not the overall result or the outcome of different categories, as all students were interviewed during the same period.

The written transcripts of each interview were read and discussed by three of the authors, one senior physician who teaches undergraduate medical students, one medical student and one graduate physician who was very familiar with phenomenography. Phenomenography focuses on the interaction between the population (the students) and the phenomenon (conception of learning). The researcher must therefore inhibit any pre-existing relationship between him or herself and the phenomenon. The basic idea of the phenomenographic approach is to identify and describe individual conceptions of some aspect of reality as faithfully as possible.^33^ We therefore believe that the different background of the researchers was an advantage when analyzing the interviews.

## Conclusions

No major difference was observed in the student's conception of learning in the two different settings; one with separated subject-specific written examinations and one with an integrated written final examination. Comprehensive learning was the most common learning approach and students who changed during the course most often switched to this in both settings. In the comprehensive learning the student is eager to learn, but applies a superficial learning approach, memorizing disintegrated data without seeking in-depth understanding of their relationship. We also observed that passing the examination rather than learning becomes the most important goal of studying, as the examination gets closer. The implication of the study for medical education is that an integrated examination is not enough to change the medical student's conception of learning for a written examination.

### Acknowledgements

Lars Owe Dahlgren, deceased Professor at the University of Linkoping, is gratefully acknowledged for valuable advice and fruitful discussions in the initial part of the study. Associate Professor Jonas Hedlund, course director in infectious diseases, and Professor Jan Ostergren, former course director in internal medicine, both at the Department of Medicine Solna, Karolinska Institutet, are thanked for good collaboration. This study was supported with grants from the Stockholm County Council and the Karolinska Institutet.

### Conflict of interest

The authors declare that they have no conflict of interest.

## References

[1] Jones R, Higgs R, de Angelis C, Prideaux D (2001). Changing face of medical curricula.. Lancet.

[2] ALDERSON JC, WALL D (1993). Does Washback Exist?. Applied Linguistics.

[3] Calman KC. Medical education. Past, present, and future: handing on learning. Edinburgh: Churchill Livingstone; 2007.

[4] Entwistle NJ, Entwistle A (1991). Contrasting forms of understanding for degree examinations: the student experience and its implications.. High Educ.

[5] Gibbs G, Simpson C. Conditions under which assessment supports students' learning. Learn Teach High Educ. 2004;1(1):3-31.

[6] Marton F, Saljo R. Approaches to learning. In: Marton F, Housell D, Entwistle N, editors. The experience of learning: implications for teaching and studying in higher education (2nd Ed). Edinburgh: Scottish Academic Press; 1997.

[7] Ramsden P. Learning to teach in higher education (2nd ed). London: Routledge Falmer; 2003.

[8] Reid WA, Duvall E, Evans P (2007). Relationship between assessment results and approaches to learning and studying in Year Two medical students.. Med Educ.

[9] McManus IC (1995). Examining the educated and the trained.. Lancet.

[10] Baerheim A, Meland E (2003). Medical students proposing questions for their own written final examination: evaluation of an educational project.. Med Educ.

[11] McManus IC, Richards P, Winder BC, Sproston KA (1998). Clinical experience, performance in final examinations, and learning style in medical students: prospective study.. BMJ.

[12] Wilhelmsson N, Bolander-Laksov K, Dahlgren LO, Hult H, Nilsson G, Ponzer S (2013). Long-term understanding of basic science knowledge in senior medical students.. Int J Med Educ.

[13] Marton F, Saljo R. On qualitative differences in learning: I. Outcome and process. Br J Educ Psychol. 1976;46(1):4-11.

[14] Marton F, Saljo R. On qualitative differences in learning: II. Outcome as a function of the learner's conception of the task. Br J Educ Psychol. 1976;46(2):115-27.

[15] Lonka K, Lindblom-Ylanne S (1996). Epistemologies, conceptions of learning, and study practices in medicine and psychology.. High Educ.

[16] Van der Veken J, Valcke M, De Maeseneer J, Derese A (2009). Impact of the transition from a conventional to an integrated contextual medical curriculum on students' learning patterns: a longitudinal study.. Med Teach.

[17] Vermetten YJ, Lodewijks HG, Vermunt JD (1999). Consistency and variability of learning strategies in different university courses.. High Educ.

[18] Evans CJ, Kirby JR, Fabrigar LR (2003). Approaches to learning, need for cognition, and strategic flexibility among university students.. Br J Educ Psychol.

[19] Marton F (1981). Phenomenography ? Describing conceptions of the world around us.. Instr Sci.

[20] Marton F, Booth S. Learning and awareness. Mahwah, NJ: Lawrence Erlbaum Associates Inc; 1997.

[21] Wahlström R, Dahlgren LO, Tomson G, Diwan VK, Beermann B (1997). Changing Primary Care Doctors' Conceptions - A Qualitative Approach to Evaluating an Intervention.. Adv Health Sci Educ Theory Pract.

[22] Dahlgren MA, Hult H, Dahlgren LO, af Segerstad HH, Johansson K (2006). From senior student to novice worker: learning trajectories in political science, psychology and mechanical engineering.. Studies in Higher Education.

[23] Vermunt JD, Vermetten YJ (2004). Patterns in Student Learning: Relationships Between Learning Strategies, Conceptions of Learning, and Learning Orientations.. Educational Psychology Review.

[24] Patel VL, Yoskowitz NA, Arocha JF (2009). Towards effective evaluation and reform in medical education: a cognitive and learning sciences perspective.. Adv Health Sci Educ Theory Pract.

[25] Entwistle NJ, Ramsden P. Understanding student learning. London: Croom Helm; 1983.

[26] Chin C, Brown DE. Learning in science: a comparison of deep and surface approaches. J Res Sci Teach. 2000;37(2):109-38.

[27] Morse JM, Field P-A. Qualitative research methods for health professionals (2nd Ed). Thousand Oaks, CA: SAGE Publications Inc; 1995.

[28] Polit DF, Beck CT. Essentials of nursing research: appraising evidence for nursing practice (8th Ed). Philadelphia: Wolters Kluwer Health/Lippincott Williams & Wilkins; 2013.

[29] Vermunt JD. Metacognitive, cognitive and affective aspects of learning styles and strategies: a phenomenographic analysis. High Educ. 1996;31(1):25-50.

[30] Biggs JB, Collis KF. Evaluating the quality of learning. The SOLO taxonomy (Structure of the observed learning outcome). New York: Academic Press; 1982.

[31] Biggs J. Assumptions underlying new approaches to educational assessment: implications for Hong Kong. Curriculum Forum. 1995;4(2):1-22.

[32] Watkins D, Dahlin B, Ekholm M (2005). Awareness of the backwash effect of assessment: A phenomenographic study of the views of Hong Kong and Swedish lecturers.. Instr Sci.

[33] Sandbergh J (1997). Are Phenomenographic Results Reliable?. Higher Education Research & Development.

